# Homology Modelling and Molecular Docking Studies of Selected Substituted Benzo[*d*]imidazol-1-yl)methyl)benzimidamide Scaffolds on *Plasmodium falciparum* Adenylosuccinate Lyase Receptor

**DOI:** 10.1177/1177932219865533

**Published:** 2019-07-31

**Authors:** Gbolahan O Oduselu, Olayinka O Ajani, Yvonne U Ajamma, Benedikt Brors, Ezekiel Adebiyi

**Affiliations:** 1 Covenant University Bioinformatics Research (CUBRe), Covenant University, Ota, Nigeria; 2 Department of Chemistry, College of Science and Technology, Covenant University, Ota, Nigeria; 3 Division of Applied Bioinformatics, German Cancer Research Center (DKFZ), Heidelberg, Germany; 4 Department of Computer and Information Science, Covenant University, Ota, Nigeria

**Keywords:** ADMET prediction, malaria, benzimidazole, antimalarial activity, molecular docking, drug target, benzimidamide, in silico

## Abstract

*Plasmodium falciparum* adenylosuccinate lyase (*Pf*ADSL) is an important enzyme in purine metabolism. Although several benzimidazole derivatives have been commercially developed into drugs, the template design as inhibitor against *Pf*ADSL has not been fully explored. This study aims to model the 3-dimensional (3D) structure of *Pf*ADSL, design and predict in silico absorption, distribution, metabolism, excretion and toxicity (ADMET) of 8 substituted benzo[*d*]imidazol-1-yl)methyl)benzimidamide compounds as well as predict the potential interaction modes and binding affinities of the designed ligands with the modelled *Pf*ADSL. *Pf*ADSL 3D structure was modelled using SWISS-MODEL, whereas the compounds were designed using ChemDraw Professional. ADMET predictions were done using OSIRIS Property Explorer and Swiss ADME, whereas molecular docking was done with AutoDock Tools. All designed compounds exhibited good in silico ADMET properties, hence can be considered safe for drug development. Binding energies ranged from −6.85 to −8.75 kcal/mol. Thus, they could be further synthesised and developed into active commercial antimalarial drugs.

## Introduction

**Figure 1.**
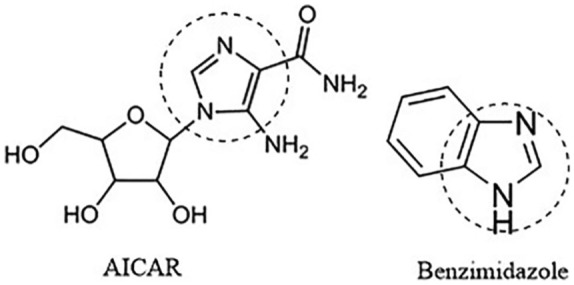
Structures of AICAR and benzimidazole showing the regions of similarity. Abbreviation: AICAR, 5-aminoimidazole-4-carboxamide ribonucleotide.

Malaria is one of the most challenging infectious diseases to eradicate, especially in Sub-Saharan Africa.^1^ *Plasmodium falciparum* remains the most prevalent malaria parasite in the world accounting for 216 million estimated cases in 2016.^2^ The drug resistance of malaria parasite has led to the need and search for new chemical scaffolds that have novel modes of action and can act through new protein targets.^3,4^ One of such protein targets in *P falciparum* is the adenylosuccinate lyase (ADSL), which is an important enzyme in purine metabolism.^5^ The de novo purine biosynthetic pathway that gives rise to the formation of adenosine monophosphate (AMP), catalysed by ADSL, is absent in *P falciparum*, making it a potential drug target for antimalarial studies.^6,7^ Cassera et al^7^ reported that 5-aminoimidazole-4-carboxamide ribonucleotide (AICAR) and its analogues can serve as potential inhibitors for ADSL of *P falciparum*, hence novel putative antiparasitic agents. Benzimidazole derivatives (substituted benzo[*d*]imidazol-1-yl)methyl)benzimidamides) were considered as potential analogues for AICAR due to similarities in chemical structure (Figure 1), and could be evaluated for their antimalarial propensity. Benzimidazole derivatives have been widely used in recent years due to their wide range of pharmacological activities including antimalarial,^8^ antileishmanial,^9^ analgesics,^10^ anticancer,^11^ antitumour,^12^ antimicrobial,^13^ anti-inflammatory,^14^ antihepatitis C virus,^15^ antihelmintic,^16^ antibacterial^17^ and antitrypanosomal^18^ activities. Although several benzimidazole derivatives have been synthesised and developed into commercially available drugs, little is known about the design of the template as an inhibitor against *P falciparum* ADSL (*Pf*ADSL).

Over the years, different approaches have been used to improve how antimalarial agents are designed, put through clinical trials and eventually released as commercially available drugs.^19^ One of such approaches is structure-based drug design (SBDD), which relies on the knowledge of the 3-dimensional (3D) structure of the protein target to design a suitable ligand that can function as its potential inhibitor.^20^ In a situation where the experimental 3D structure of the protein is not available, homology model can be built from its amino acid sequence.^21^ Molecular docking is an important technique in SBDD, which can be applied in facilitating and speeding up the development of antimalarial agents or drugs that can be active against the deadly malaria parasite.^22^ Molecular docking has helped scientists to virtually screen a library of ligands (or compounds) against a target protein and predict the binding conformations and affinities of the ligands to the target.^19^ The aim of this study is to model the 3D structure of *Pf*ADSL, design and predict the in silico absorption, distribution, metabolism, excretion and toxicity (ADMET) of some substituted benzo[*d*]imidazol-1-yl)methyl)benzimidamide compounds as well as predict the potential interaction modes and binding affinities of the designed ligands with the modelled *Pf*ADSL.

## Materials and Methods

### Homology modelling of PfADSL and the target-template sequence alignment

The experimental crystal structure of *Pf*ADSL is not available in the Protein Data Bank (PDB);^23^ hence, its 3D structure was modelled. The protein ID of the target (*P falciparum* adenylosuccinate lyase 3D7 strain) was retrieved from UniProt Knowledgebase (UniProtKB)^24^ with the accession number Q7KWJ4. Afterwards, the protein ID was submitted to SWISS-MODEL^25^ web server to develop a model with sufficient query sequence coverage and sequence identity. The most reliable 3D structure was selected based on the Global Model Quality Estimation (GMQE)^26^ and Qualitative Model Energy Analysis (QMEAN)^27^ values. The GMQE values are usually between 0 and 1, and the higher the number, the higher the reliability of the predicted structure, while for QMEAN, a value below 4.0 shows reliability.^28^ The similarity identity between the amino acid sequences of the homology model of *Pf*ADSL and the template structure used for the homology model were confirmed using Clustal Omega version 1.2.1.^29^

### Structure validation of modelled protein

The SWISS-MODEL web server automatically calculates the QMEAN scoring function for the estimation of the local and the global model quality based on the geometry, the interactions and the solvent potential of the protein model. It also provides the z-score ranging from 0 to 1, which are compared with the expected value for any structure. PROCHECK was used to check for the quality of the modelled 3D structure of *Pf*ADSL generated via SWISS-MODEL. For this structure validation, the .pdb file format of the modelled *Pf*ADSL was uploaded on the PDBsum web server^30^ of European Bioinformatics Institute. The .pdb file format of the modelled *Pf*ADSL was uploaded on the server to obtain both the Ramachandran plot and the Ramachandran plot statistics. While the Ramachandran plot is used in accessing the quality of a modelled protein or an experimental structure, the Ramachandran plot statistics provides information on the total number of amino acid residues found in the favourable, allowed and disallowed regions.^31^ Also, Verify3D^32^ was used to validate the structure of the modelled protein, determine how compatible a 3D structure is to its own amino acids and compare the result with that of good-known structures.

### Alignment of the PfADSL model and the template structure

The alignment of the *Pf*ADSL model and template structure was carried out using PyMOL molecular viewer^33^ to show how closely related the carbon atoms are. This is derived from the root mean square deviation (RMSD) between the positioning of the carbon atoms of both the template and the model that is obtained from the alignment. The lower the RMSD (w.r.t 0), the more closely related the structures are.

### Ligand modelling

**Scheme 1.**
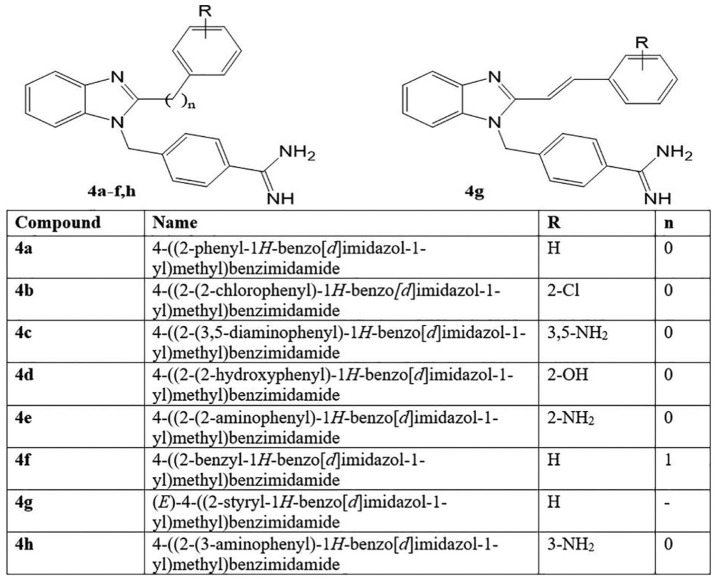
Schematic representation of the compounds **4a-h**. ‘R’ stands for substituents on the compounds; ‘n’ stands for number of CH_2._

AICAR analogues are good inhibitors of *Pf*ADSL^7^ and similar to benzimidazole, as shown in Figure 1. Therefore, the benzimidazole derivatives were built as ligands to function as potential inhibitors of *Pf*ADSL, which is the target protein. The 2-dimensional (2D) structures of the substituted benzo[*d*]imidazol-1-yl)methyl)benzimidamide compounds, **4a-h** (Scheme 1), were built using ChemDraw Professional 15.0 by PerkinElmer USA. Also, ChemDraw was used to generate the simplified molecular-input line-entry system (SMILES) that were converted to their corresponding 3D structures using FRee Online druG 3D conformation generator (FROG)^34^ and saved in .pdb format. In addition, OpenBabel software^35^ was used to convert the .pdb files to the AutoDock docking format (.pdbqt), which was further used for the docking simulation.

### In silico drug-likeness and toxicity predictions

Drug-likeness is a prediction that determines whether a particular pharmacological agent has properties consistent with being an orally active drug.^36^ This prediction is based on an already established concept by Lipinski et al,^37^ called Lipinski rule of five. The rule predicts that there is likely to be poor absorption or permeation when a compound possesses more than 5H-bond donors, 10H-bond acceptors, molecular weight greater than 500 and the calculated LogP (CLogP) greater than 5.^37^ The selection of compounds as drug candidates is also determined by a parameter called drug score.^38^ The higher the drug score value, the higher the chance of the compound being considered as a drug candidate.^38^ The in silico drug-likeness and toxicity predictions of the designed ligands were carried out using OSIRIS Property Explorer^39^ and Swiss ADME predictor.^40,41^ OSIRIS Property Explorer programme estimates the mutagenic, tumorigenic, irritant and reproductive risks, and also provides information on the compound’s hydrophilicity (LogP), solubility (LogS), molecular weight, drug-likeness and drug score.^42^ Meanwhile, SwissADME predictor provides information on the numbers of hydrogen donors, hydrogen acceptors and rotatable bonds, total polar surface area and the synthetic accessibility of the compounds. The ligands were also subjected to Lipinski et al,^37^ Muegge et al,^43^ Ghose et al,^44^ Egan et al^45^ and Veber et al^46^ screenings using SwissADME predictor. The analyses of the compounds were compared with that of chloroquine, and only compounds without violation of any of the screenings were used for the molecular docking analysis.

### Protein preparation

The homology modelled 3D structure of the target protein, *Pf*ADSL, was downloaded from SWISS-MODEL in its .pdb format. The modelled protein structure was defined as receptor while the complexed ligands were removed using Chimera software.^47^ Furthermore, the protein was prepared by the computation of Gasteiger charges, with the addition of polar hydrogens and merging of the nonpolar hydrogens using AutoDockTools 1.5.6.^48^

### Prediction of active sites in the modelled protein

The Computed Atlas of Surface Topography of proteins (CASTp) 3.0^49^ was used to predict the active sites that were present in the modelled protein structure. CASTp is an online server that is applied in the identification and measurement of voids on 3D protein structures.^50^ The modelled 3D protein was submitted on the server, and the necessary amino acids for binding interactions were predicted.^50^

### Molecular docking analysis

It has been reported that ADSL enzymes, which were used for the docking analyses, are biologically active as homotetramers.^51,52^ The molecular docking studies were carried out using AutoDockTools, which is a free graphic user interface (GUI) for the AutoDock4.2 programme.^53^ The grid box was constructed using 58, 58, and 40, pointing in x, y, and z directions, respectively, with a grid point spacing of 0.508 Å. The centre grid box is of 14.527 Å, 56.689 Å and −5.122 Å around Arg 17A, Tyr 18A, Asn 312A, His 173C, Asn 90D, Asp 92D, Gln 250D, Arg 338D, Ser 343D and Arg 347D. These amino acids were selected based on the CASTp result and the alignment of the modelled 3D structure to the template structure. In addition, the docking analysis was executed using Lamarckian Genetic Algorithm 4.2, and the macromolecule was kept rigid throughout the docking simulation. The number of genetic algorithm runs was set at 10, and the other docking parameters were left at default values. Ten different conformations were generated for each ligand scored using AutoDock 4.2 scoring functions and were ranked according to their binding energies. AutoDockTools, PyMOL and LigPlot^54^ were used for the post-docking analyses.

## Results and Discussion

### Homology modelling of PfADSL and the target-template sequence alignment

A 3D structure of *Pf*ADSL was built using SWISS-MODEL with GMQE of 0.80 and QMEAN of −1.46. Also, *Plasmodium vivax* ADSL Pv003765 with AMP bound (PDB ID: 2QGA; resolution: 2.01 Å)^55^ was identified to have the closest template to *Pf*ADSL with a similarity identity of 63.91% and sequence similarity of 0.50. The GMQE value of 0.80 and QMEAN score of −1.46 indicate that the modelled structure is reliable and has a good quality.^26,28^

**Figure 2.**
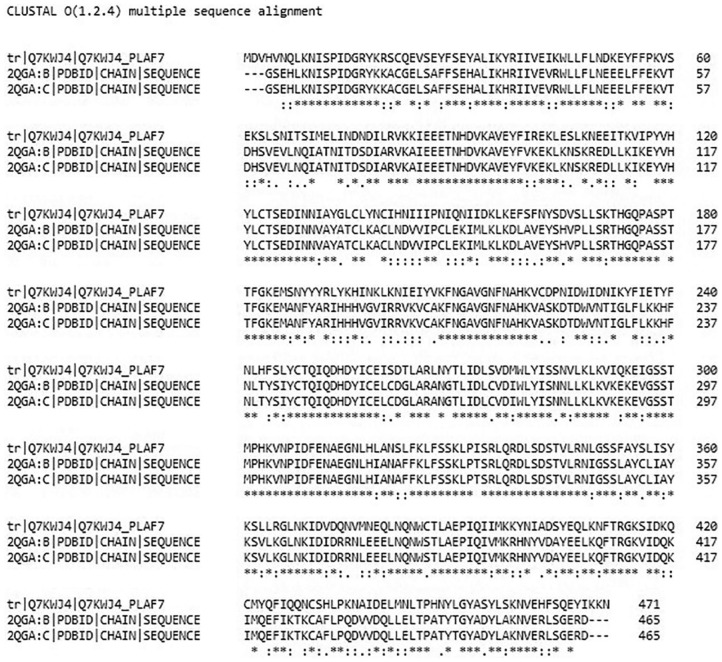
Alignment of the amino acid sequences of *Plasmodium falciparum* ADSL and the crystal structure of 2QGA. Abbreviation: ADSL, adenylosuccinate lyase. ‘*’ represents positions that have single, fully conserved residue; ‘:’ indicates conservation between groups of strongly similar properties; ‘.’ indicates conservation between groups of weakly similar properties.

The multiple sequence alignment of the amino acid sequences^56^ of the *Pf*ADSL (UniProtKB ID: Q7KWJ4) and *P vivax* ADSL with AMP bound (PDB ID: 2QGA) is shown in Figure 2. A percentage identity matrix of 63.36% was obtained, which confirms the similarity identity of 63.91% obtained from the homology modelling.

### Structure validation of modelled protein

**Figure 3.**
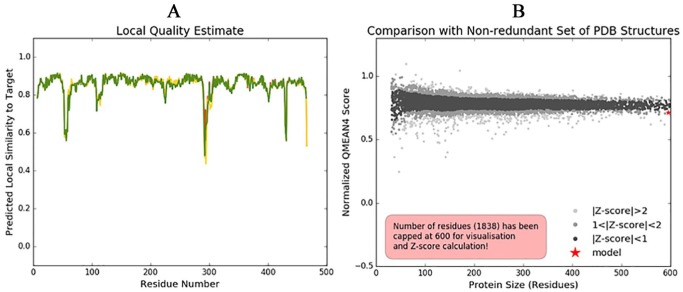
Structure validation of modelled *Pf*ADSL: (A) Local quality estimate of the residues of the predicted *Pf*ADSL model; (B) comparison of the predicted *Pf*ADSL structure with nonredundant set of PDB structures. Abbreviation: *Pf*ADSL, *Plasmodium falciparum* adenylosuccinate lyase, PDB, Protein Data Bank.

The plot of the predicted local similarity to target against the residue number of the predicted 3D structure of the modelled protein was graphically represented (Figure 3A). The value of most of the residues was close to 1, indicating that the local quality estimate of the residues of the predicted model is good. The residues with values lower than 0.6 were considered to be of low quality. The modelled protein structure also lies within the range of other protein structures in PDB, which confirms its reliability (Figure 3B).

**Figure 4.**
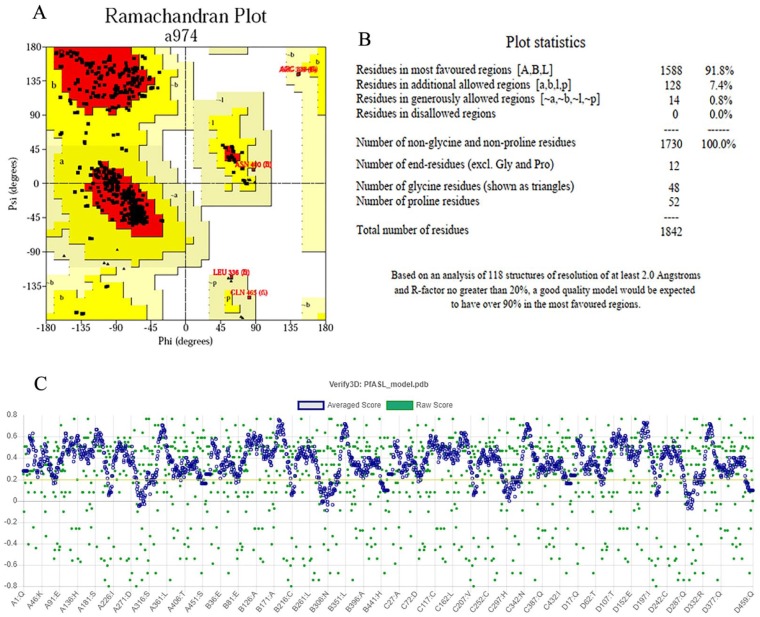
Structure validation using (A) Ramachandran plot; (B) Ramachandran plot statistics of the homology modelled *Pf*ADSL; and (C) Verify3D. Abbreviation: *Pf*ADSL, *Plasmodium falciparum* adenylosuccinate lyase.

Both the Ramachandran plot (Figure 4A) and the Ramachandran plot statistics (Figure 4B) were obtained from PDBsum web server. The Ramachandran plot statistics implied that the modelled 3D structure of *Pf*ADSL has 91.8% of its residues in the most favoured regions, 7.4% of its residues in additional allowed regions, 0.8% of its residues in the generously allowed regions and 0.0% of its residues in disallowed regions of the Ramachandran plot. This also validates that the modelled 3D structure is a good quality model. Also, the Verify3D plot of the modelled protein (Figure 4C) was obtained for the structure validation and it showed as PASS. The 3D environment profile shows that 85.64% of the residues have averaged 3D-1D score ⩾ 0.2, which suggests the validity of the modelled protein.

### Alignment of the PfADSL model and template (2GQA) structure

**Figure 5.**
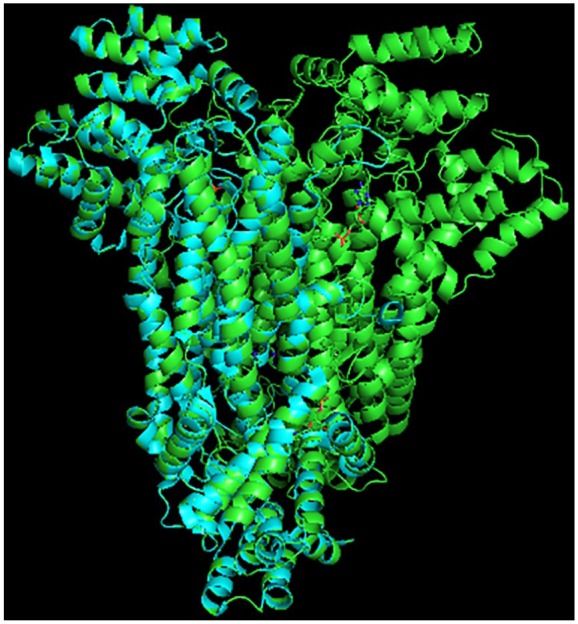
Alignment of the *Pf*ADSL model and the 2GQA template structure of *Plasmodium vivax* ADSL. Abbreviation: *Pf*ADSL, *Plasmodium falciparum* adenylosuccinate lyase.

A RMSD value of 0.105 Å was obtained from the alignment computed using PyMOL molecular viewer, indicating that the structures were closely related (Figure 5). The template structure is represented by the blue helices, whereas the protein model is represented by the green helices. The alignment showed that the chain B and chain C of the dimer template structure (2QGA) corresponded to the chain C and chain D of the tetramer structure of the protein model. Meanwhile, it was observed from the molecular viewer that the binding of the AMP to the amino acid residues of 2QGA at His 168B, Asn 85C, Asp 87C, Gln 245C, Ser 338C, Arg 333C and Arg 342D also corresponded with the binding of the AMP with the amino acid residues of the modelled template at His 173C, Asn 90D, Asp 92D, Gln 250D, Ser 343D, Arg 338D and Arg 347D.

### In silico results of risks and drug-likeness of ligands

*a. OSIRIS property explorer result*. With the exception of compound **4c**, all the predicted toxicity risk factors for the 8 designed substituted benzo[*d*]imidazol-1-yl)methyl)benzimidamides were low (Table 1). Also, the 8 compounds had molecular weights less than 500, which implied that they are likely to be absorbed and are able to reach the place of action when administered as drugs.^57^ All compounds including the standard drug (chloroquine) had LogP values not higher than 5, suggesting good absorption and permeation across cell membranes.^57^ Among the compounds, 4-((2-benzyl-1H-benzo[*d*]imidazol-1-yl)methyl)benzimidamide **4f** had the highest value of drug score (0.83), which is higher than that of chloroquine (0.25). This high value could be as a result of the aliphatic carboxylic acid (phenyl acetic acid) on position 2 of the benzimidazole in **4f**. In general, the drug score values of compounds **4a-h** (0.3-0.83) were bigger than that of chloroquine. In addition, it was predicted that all the designed compounds possessed low mutagenic, tumorigenic, irritant and reproductive effective toxicity risks except **4c**, which was predicted to have a high mutagenic toxicity risk. However, the removal of the amino group on position 5 of the benzoic acid as seen in **4h** reduced the risk of the mutagenic toxicity of **4c**.

**Table 1.** Physicochemical properties and toxicity risks of compounds **4a-h** in comparison with chloroquine as predicted using OSIRIS Property Explorer.

Compounds	Physicochemical properties					Toxicity risks			
	Molecular weight	cLogP	Solubility prediction	Drug likeness	Drug score	Mutagenic	Tumorigenic	Irritant	Reproductive effective
**4a**	326	3.08	−3.69	4.09	0.78	Low	Low	Low	Low
**4b**	360	3.69	−4.43	3.68	0.67	Low	Low	Low	Low
**4c**	356	1.73	−3.85	−1.17	0.30	High	Low	Low	Low
**4d**	342	2.74	−3.40	3.14	0.80	Low	Low	Low	Low
**4e**	341	2.40	−3.77	0.93	0.69	Low	Low	Low	Low
**4f**	340	3.14	−2.54	3.63	0.83	Low	Low	Low	Low
**4g**	352	3.53	−2.97	2.11	0.75	Low	Low	Low	Low
**4h**	341	2.40	−3.77	1.36	0.72	Low	Low	Low	Low
**Chloroquine**	319	4.01	−4.06	7.39	0.25	High	Low	High	Low

### b. SwissADME prediction

The numbers of hydrogen bond acceptors (NHA) and hydrogen bond donors (NHD) in compounds **4a-h** (Table 2) are in accordance with the rule of five by Lipinski et al.^37^ The LogS prediction of −5.11 to −3.81 indicated that all the compounds were moderately soluble. Also, the highest value (2.89) of synthetic accessibility was recorded for compound **4g**, suggesting that it will be the most difficult to synthesise from the compound library. This could be due to the presence of a double bond in position 2 of the benzimidazole. Generally, the synthetic accessibility of all the compounds (2.44-2.89) was within the range of easy synthetic accessibility. It is also interesting to note that none of the compounds violated the Lipinski rule of five, Ghose filter, Veber rule, Egan rule and Muegge rule. This shows that all the ligands can be considered as good lead compounds in drug design.

**Table 2.** ADME prediction of compounds **4a-h** in comparison with chloroquine, predicted by SwissADME.

Compounds	Formula	NHD	NHA	NRB	TPSA (Å^2^)	LogP (iLOGP)	LogS (ESOL)	Synthetic accessibility
**4a**	C_21_H_18_N_4_	2	2	4	67.69	2.27	−4.53	2.44
**4b**	C_21_H_17_ClN_4_	2	2	4	67.69	2.11	−5.11	2.54
**4c**	C_21_H_20_N_6_	4	2	4	119.73	1.38	−3.81	2.74
**4d**	C_21_H_18_N_4_O	3	3	4	87.92	2.11	−4.38	2.52
**4e**	C_21_H_19_N_5_	3	2	4	93.71	2.00	−4.17	2.62
4f	C_22_H_20_N_4_	2	2	5	67.69	2.11	−4.17	2.62
**4g**	C_23_H_20_N_4_	2	2	5	67.69	2.34	−4.98	2.89
**4h**	C_21_H_19_N_5_	3	2	4	93.71	1.81	−4.17	2.60
**Chloroquine**	C_18_H_26_ClN_3_	1	2	8	28.16	3.95	−4.55	2.76

Abbreviations: ADME, absorption, distribution, metabolism, excretion; NHA, no. of hydrogen bond acceptors; NHD, no. of hydrogen bond donors; NRB, no. of rotatable bonds; TPSA, total polar surface area.

### Active site identification

**Figure 6.**
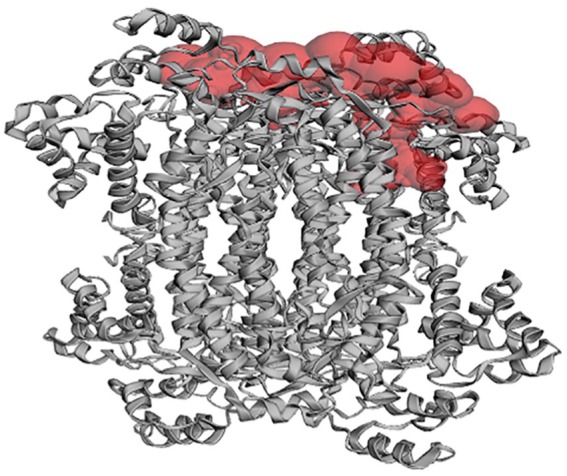
The surface of the binding pocket of the modelled protein as computed using CASTp 3.0.

From the active site prediction, a pocket was identified with an area (SA) of 2919.055 and a volume (SA) of 2797.556 (Figure 6). A total of 166 amino acid residues were predicted to be the active sites for the modelled protein. However, the following were chosen as the more favourable sites for the docking analyses due to the similarities observed from the alignment of the modelled structure to the template structure: Arg 17A, Tyr 18A, Asn 312A, His 173C, Asn 90D, Asp 92D, Gln 250D, Arg 338D, Ser 343D, Arg 347D.

### Molecular docking results

**Figure 7.**
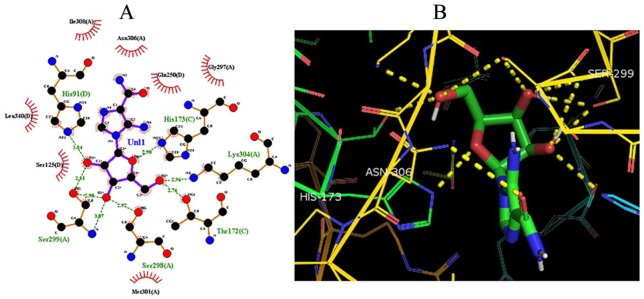
Molecular docking interactions between AICAR and the binding sites of *Pf*ADSL: (A) 2D model of the interactions between AICAR and *Pf*ADSL; (B) 3D model of the interactions between AICAR and the binding sites of *Pf*ADSL. Abbreviation: AICAR, 5-aminoimidazole-4-carboxamide ribonucleotide; *Pf*ADSL, *Plasmodium falciparum* adenylosuccinate lyase.

**Figure 8.**
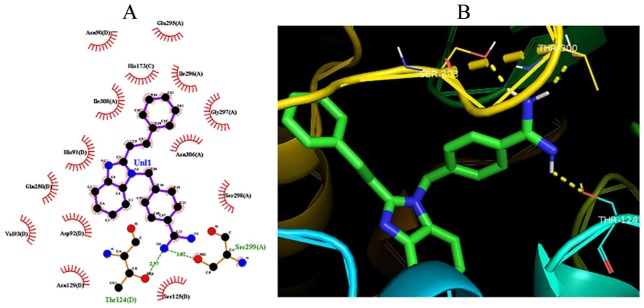
Molecular docking interactions between **4g** and the binding sites of *Pf*ADSL: (A) 2D model of the interactions; (B) 3D model of the interactions. Abbreviation: *Pf*ADSL, *Plasmodium falciparum* adenylosuccinate lyase.

The obtained binding energies and hydrogen bonds of compounds **4a-h** from the molecular docking simulations are detailed in Table 3, whereas the docked conformation of AICAR and (*E*)-4-((2-styryl-1H-benzo[d]imidazol-1-yl)methyl)benzimidamide (**4g**) in the active sites of *Pf*ADSL is presented in Figures 7 and 8, respectively. Structure–activity relationship studies based on the observed dock score values of the compounds suggest that the presence of amidine group, RC(=NH)-NH_2_, on the compounds could be responsible for the low binding energies and strong binding affinity (Table 3). Also, the presence of amino group on position 2 of the substituted phenyl in **4e** (–7.48 kcal/mol) increased the binding affinity of the compound as against it being on position 3 as observed in **4h** (–7.20 kcal/mol). The di-substitution of amino groups on positions 3 and 5 of the substituted phenyl in **4e** did not have a positive impact on the binding energy but rather reduced its binding affinity (–6.85 kcal/mol) as against the mono-substitutions on position 2 of **4e** and position 3 on **4h**.

**Table 3.** Energy-based interactions and hydrogen bonds for benzimidazole derivatives **4a-h**, AICAR and AMP docked into modelled *Pf*ADSL.

Compounds	Binding energies (Kcal/mol)	Hydrogen bonds and the bond lengths
**4a**	−7.52	Ser 298A (3.01 Å), Ser 299A (3.19 Å)
**4b**	−7.85	Ser 298A (3.17 Å), Ser 299A (2.98 Å)
**4c**	−6.85	Asn 90D (2.90 Å), Thr 124D (2.90 Å), Thr 300A (2.67 Å)
**4d**	−7.03	Ser 298A (2.87 Å), Ser 299A (3.10 Å), Thr 124D (2.79 Å)
**4e**	−7.48	Gln 250D (3.05 Å), Ile 296A (2.71 Å)
**4f**	−8.09	Glu 295A (2.46 Å), Asn 306A (2.70 Å)
**4g**	−8.75	Ser 299A (3.02 Å), Thr 124D (2.97 Å)
**4h**	−7.20	Ser 298A (3.17 Å), Ser 299A (3.25 Å), Asp 92D (2.77 Å)
AICAR	−5.49	Ser 298A (2.92 Å), Ser 299A (2.81 Å), His 91D (3.04 Å), Thr 172 C (2.76 Å), Lys 304A (2.96 Å), His 173C (2.90 Å)
AMP	−5.10	Tyr 18A (2.56 Å), Asn 312A (3.04 Å), Arg 17A (2.94 Å), Asn 90D (2.72 Å), Gln 250D (2.72 Å), Arg 338D (3.01 Å), Ser 343D (2.95 Å), Arg 347 (2.76 Å)

Abbreviations: AICAR, 5-aminoimidazole-4-carboxamide ribonucleotide; AMP, adenosine monophosphate.

Furthermore, all the designed compounds exhibited dock score values between −6.85 and −8.75 kcal/mol, having lower binding energies than that of the complexed ligand (AMP) that had a binding energy of −5.10 kcal/mol. Also, the binding energies of the compounds were lower than AICAR (–5.49 kcal/mol), which has been reported to be a potential inhibitor of *Pf*ADSL.^7^ The lowest autodock score and the best interactions were used to ascertain the compound with the best conformation.^2^ The best dock score among the designed benzimidazole derivatives was −8.75 kcal/mol for compound **4g**. The hydrogen bond formed between compound **4g** and the amino acid residues (Ser 299A, Thr 124D) of *Pf*ADSL also validates the functional and structural stability of the ligand-protein complex.^2^ Thus, the binding model reported in this study suggests that these substituted benzo[*d*]imidazol-1-yl)methyl)benzimidamides behave as *Pf*ADSL inhibitors and show some key structural points to be considered in future optimization.

## Conclusion

*Pf*ADSL is a potential drug target that can be considered in the design of antimalarial compounds to combat the malaria menace. This study gives an insight into the design and prediction of potential interaction modes and binding affinities of 8 substituted benzo[*d*]imidazol-1-yl)methyl)benzimidamide compounds with homology modelled *Pf*ADSL. (*E*)-4-((2-styryl-1H-benzo[*d*]imidazol-1-yl)methyl)benzimidamide, **4g**, had the highest dock score value among the designed ligands. All the designed compounds possessed good in silico ADMET properties, demonstrating their safety for further synthesis and development into active commercially available antimalarial drugs. Also, experimental characterization is needed for further validation of the protein target.
